# Supplementary material for: Estimating the effect of anticipated depression treatment-related stigma on depression remission among people with noncommunicable diseases and depressive symptoms in Malawi
Source: PLoS One. 2023 Mar 16;18(3):e0282016. doi: 10.1371/journal.pone.0282016 (PMC10019662; doi:10.1371/journal.pone.0282016)
Supplement: S1 Appendix — (PDF) [file pone.0282016.s002.pdf]

S1 Appendix. Stigma Questionnaire.

Note: All responses (except item #4) are on a 5-point Likert scale, from strongly disagree to strongly agree.

[Vignette]: *Thandi is a woman who lives in a nearby village. For the last several weeks Thandi has been feeling really down. She wakes up in the morning with a sad mood and heavy feeling that stick with her all day long. She is not enjoying things the way she normally would. In fact, nothing seems to give her pleasure. Even when good things happen, they do not seem to make Thandi happy. The smallest tasks are difficult to accomplish. She finds it hard to concentrate on anything. She feels out of energy, out of steam, and cannot do things she usually does. And even though Thandi feels tired, when night comes, she cannot go to sleep. Thandi feels pretty worthless, very discouraged, and guilty. Thandi's family has noticed that she has lost appetite and weight. She has pulled away from them and just does not feel like talking.*

1. Being around Thandi would make me feel uncomfortable.
2. Thandi should feel embarrassed about her situation.
3. Members of Thandi's family would be better off if Thandi's situation was kept secret.
4. Would you say that Thandi's situation is most likely caused by HIV, asthma, depression, schizophrenia, poverty, stress, the normal ups and downs of life, or something else? The respondent should choose only one.

[Vignette]: *Let me tell you a little bit more about Thandi. A few weeks ago, Thandi went to the clinic to see a doctor about her situation. The doctor told Thandi that she has an illness called depression. The doctor said that depression can be treated with medication and with counseling. Now I want to ask you a few more statements about Thandi. Tell me whether you agree or disagree with each one.*

5. Being around someone diagnosed with depression like Thandi would make me feel uncomfortable.
6. Thandi should feel embarrassed about her diagnosis of depression.
7. Members of Thandi's family would be better off if her diagnosis of depression was kept secret.
8. If Thandi were going to a clinic regularly to speak with a trained therapist about her problems to help treat her depression, and people found out she was going, she would lose some of her friends.
9. If Thandi were taking medication to help treat her depression, and people found out she was taking medication, she would lose some of her friends.
