# Supplementary material for: Estimating the effect of anticipated depression treatment-related stigma on depression remission among people with noncommunicable diseases and depressive symptoms in Malawi
Source: PLoS One. 2023 Mar 16;18(3):e0282016. doi: 10.1371/journal.pone.0282016 (PMC10019662; doi:10.1371/journal.pone.0282016)
Supplement: S1 Table — Data distributions reported in this table were drawn from the 1st imputed data set. Only imputed analytic variables (analytic variables that previously had missing observations) are displayed here. (PDF) [file pone.0282016.s003.pdf]

S2 Table. Post-Imputation Distribution of Analysis Variables that Required Imputation[1] (N=743).

Variable	Mean	SD
Baseline Depressive Symptoms (PHQ-9)	9.3	3.9
Baseline Anxiety Symptoms (GAD-7)	7.0	4.2
Baseline Stressful Life Events (LES)	3.0	2.3
Baseline PTSD Symptoms (PCL-C)	42.9	14.6
Baseline Social Support (MSPSS)	17.2	4.2
Baseline Stigma Sub-Scale: Treatment Stigma	1.4	1.1
Baseline Stigma Sub-Scale: Negative Affect	2.2	1.0
Baseline Stigma Sub-Scale: Disclosure Carryover	2.4	1.2
Baseline Standardized SF8 Score	0.2	0.9
Baseline Standardized Wealth Score	0.0	1.0
Adaptive Coping Behaviors (Brief COPE)	7.5	3.0
	**N**	**%**
**High Anticipated Treatment-Related Stigma at Baseline**		
No	563	76%
Yes	180	24%
**Depression Remission (PHQ9<5) at 3-month Interview**		
No	450	61%
Yes	293	39%
**Baseline Stressful Life Events (LES)**		
Employment Related	128	17%
Personal Health Related	181	24%
Family Relationship Related	214	29%
**Employment at Baseline**		
Farmer	330	44%
Business Owner	131	18%
Homemaker	143	19%
Other employment	93	13%
Not currently employed	46	6%

1. Data distributions reported in this table were drawn from the 1st imputed data set. Only imputed analytic variables (analytic variables that previously had missing observations) are displayed here.
