# Supplementary material for: Estimating the effect of anticipated depression treatment-related stigma on depression remission among people with noncommunicable diseases and depressive symptoms in Malawi
Source: PLoS One. 2023 Mar 16;18(3):e0282016. doi: 10.1371/journal.pone.0282016 (PMC10019662; doi:10.1371/journal.pone.0282016)
Supplement: S2 Table — Data distributions reported in this table were drawn from the 1st imputed data set. Only variables used in the design of the inverse probability weights are displayed here. (PDF) [file pone.0282016.s004.pdf]

S3 Table. Pre- and Post-Weight Distributions[1] of Variables Used to Design Inverse Probability of Treatment Weights (N=743).

Variable	Target Distribution[2]		Pre-Weighted Distributions					Post-Weighted Distributions				
			Low/Neutral Stigma		High Stigma		Cohen's *d*[3]	Low/Neutral Stigma		High Stigma		Cohen's *d*[3]
	Mean	SD	Mean	SD	Mean	SD		Mean	SD	Mean	SD	
Baseline Depressive Symptoms (PHQ-9)	9.30	3.9	9.15	3.7	9.77	4.5	-0.16	9.44	3.9	9.44	4.1	0.00
Baseline Social Support (MSPSS)	17.21	4.2	17.31	4.1	16.91	4.6	0.09	17.19	4.2	16.92	4.6	0.06
Baseline Standardized Wealth Score	-0.01	1.0	0.04	1.0	-0.16	1.0	0.20	-0.02	1.0	-0.09	1.0	0.08
Adaptive Coping Behaviors (Brief COPE)	7.48	3.0	7.73	2.9	6.69	3.2	0.35	7.5	3.0	7.4	3.0	0.03
Employment Related	0.17	0.4	0.19	0.4	0.12	0.3	0.18	0.17	0.4	0.19	0.4	-0.03
Personal Health Related	0.24	0.4	0.23	0.4	0.28	0.4	-0.11	0.25	0.4	0.28	0.4	-0.07
Family Relationship Related	0.29	0.5	0.29	0.5	0.27	0.4	0.05	0.29	0.5	0.29	0.5	0.01
Counseling Referral[4]	0.83	0.4	0.83	0.4	0.84	0.4	-0.05	0.83	0.4	0.84	0.4	-0.04
Urbanicity: Rural[4]	0.19	0.4	0.11	0.3	0.42	0.5	-0.85	0.19	0.4	0.19	0.4	0.00
Urbanicity: Periurban[4]	0.64	0.5	0.70	0.5	0.44	0.5	0.54	0.63	0.5	0.65	0.5	-0.03
Urbanicity: Urban[4]	0.18	0.4	0.19	0.4	0.13	0.3	0.15	0.18	0.4	0.16	0.4	0.03
Education: No Formal School[4]	0.16	0.4	0.15	0.4	0.19	0.4	-0.10	0.15	0.4	0.14	0.4	0.03
Education: Standard 1-5[4]	0.32	0.5	0.31	0.5	0.34	0.5	-0.07	0.32	0.5	0.35	0.5	-0.04
Education: Standard 6-8[4]	0.30	0.5	0.30	0.5	0.29	0.5	0.02	0.30	0.5	0.30	0.5	-0.01
Education: Secondary School[4]	0.17	0.4	0.19	0.4	0.12	0.3	0.19	0.17	0.4	0.16	0.4	0.04
Education: Postsecondary School[4]	0.05	0.2	0.05	0.2	0.06	0.2	-0.07	0.05	0.2	0.05	0.2	0.01
Employment: Farmer[4]	0.44	0.5	0.41	0.5	0.54	0.5	-0.25	0.44	0.5	0.45	0.5	-0.02
Employment: Other Employment[4]	0.13	0.3	0.13	0.3	0.11	0.3	0.08	0.13	0.3	0.11	0.3	0.04
Employment: Business Owner[4]	0.18	0.4	0.18	0.4	0.17	0.4	0.03	0.18	0.4	0.18	0.4	-0.01
Employment: Homemaker[4]	0.19	0.4	0.21	0.4	0.12	0.3	0.24	0.19	0.4	0.20	0.4	-0.02
Employment: Not Currently Employed[4]	0.06	0.2	0.06	0.2	0.07	0.3	-0.03	0.07	0.3	0.06	0.2	0.04

1. Data distributions reported in this table were drawn from the 1st imputed data set. Only variables used in the design of the inverse probability weights are displayed here.
2. Inverse probability of treatment (IPT) weights estimate the average treatment effect (ATE) and target the distribution of variables in the overall sample.
3. Cohen's *d* represents the standardized difference between the mean value in the low/neutral stigma group and the high stigma group. The difference was calculated as $\frac{\bar{x}_{low}-\bar{x}_{high}}{s_{pooled}}$.[69,70]
4. These variables were represented as binaries; mean values therefore represent the percentage of participants belonging to this category.
