# Supplementary material for: Estimating the effect of anticipated depression treatment-related stigma on depression remission among people with noncommunicable diseases and depressive symptoms in Malawi
Source: PLoS One. 2023 Mar 16;18(3):e0282016. doi: 10.1371/journal.pone.0282016 (PMC10019662; doi:10.1371/journal.pone.0282016)
Supplement: S3 Table — Data distributions reported in this table were drawn from the 1st imputed data set. Only variables used in the design of the inverse probability weights are displayed here. (PDF) [file pone.0282016.s005.pdf]

S4 Table. Post-Weight Distributions[1] After Applying Inverse Probability Weights to Estimate the Average Treatment Effect in the Treated[2] (N=743).

Variable	Target Distribution[2]		Post-Weighted Distributions				
			Low/Neutral Stigma		High Stigma		
	Mean	SD	Mean	SD	Mean	SD	Cohen's *d*[3]
Baseline Depressive Symptoms (PHQ-9)	9.77	4.5	10.31	4.6	9.77	4.5	0.12
Baseline Social Support (MSPSS)	16.91	4.6	16.82	4.6	16.91	4.6	-0.02
Baseline Standardized Wealth Score	-0.16	1.0	-0.2	1.0	-0.16	1.0	-0.04
Adaptive Coping Behaviors (Brief COPE)	6.69	3.2	6.85	3.0	6.69	3.2	0.05
Employment Related	0.12	0.3	0.13	0.3	0.12	0.3	0.04
Personal Health Related	0.28	0.4	0.29	0.5	0.28	0.4	0.02
Family Relationship Related	0.27	0.4	0.28	0.4	0.27	0.4	0.02
Counseling Referral[4]	0.84	0.4	0.83	0.4	0.84	0.4	-0.03
Urbanicity: Rural[4]	0.42	0.5	0.28	0.4	0.42	0.5	0.01
Urbanicity: Periurban[4]	0.44	0.5	0.44	0.5	0.44	0.5	0.00
Urbanicity: Urban[4]	0.13	0.3	0.13	0.3	0.13	0.3	-0.01
Education: No Formal School[4]	0.19	0.4	0.17	0.4	0.19	0.4	-0.06
Education: Standard 1-5[4]	0.34	0.5	0.36	0.5	0.34	0.5	0.04
Education: Standard 6-8[4]	0.29	0.5	0.29	0.5	0.29	0.5	0.00
Education: Secondary School[4]	0.12	0.3	0.11	0.3	0.12	0.3	-0.01
Education: Postsecondary School[4]	0.06	0.2	0.07	0.3	0.06	0.2	0.04
Employment: Farmer[4]	0.54	0.5	0.51	0.5	0.54	0.5	-0.05
Employment: Other Employment[4]	0.11	0.3	0.11	0.3	0.11	0.3	0.00
Employment: Business Owner[4]	0.17	0.4	0.18	0.4	0.17	0.4	0.03
Employment: Homemaker[4]	0.12	0.3	0.11	0.3	0.12	0.3	-0.02
Employment: Not Currently Employed[4]	0.07	0.3	0.09	0.3	0.07	0.3	0.08

1. Data distributions reported in this table were drawn from the 1st imputed data set. Only variables used in the design of the inverse probability weights are displayed here.
2. The inverse probability weights used here were designed to estimate the average treatment effect in the treated (ATT) and target the distribution of variables in the high stigma group. The pre-weight distributions of the low/neutral and high stigma groups are shown in Supplementary Table 3.
3. Cohen's *d* represents the standardized difference between the mean value in the low/neutral stigma group and the high stigma group. The difference was calculated as $\frac{\bar{x}_{low}-\bar{x}_{high}}{s_{pooled}}$.[69,70]
4. These variables were represented as binaries; mean values therefore represent the percentage of participants belonging to this category.
