# Supplementary figures and images for: Estimating the effect of anticipated depression treatment-related stigma on depression remission among people with noncommunicable diseases and depressive symptoms in Malawi
Source: PLoS One. 2023 Mar 16;18(3):e0282016. doi: 10.1371/journal.pone.0282016 (PMC10019662; doi:10.1371/journal.pone.0282016)

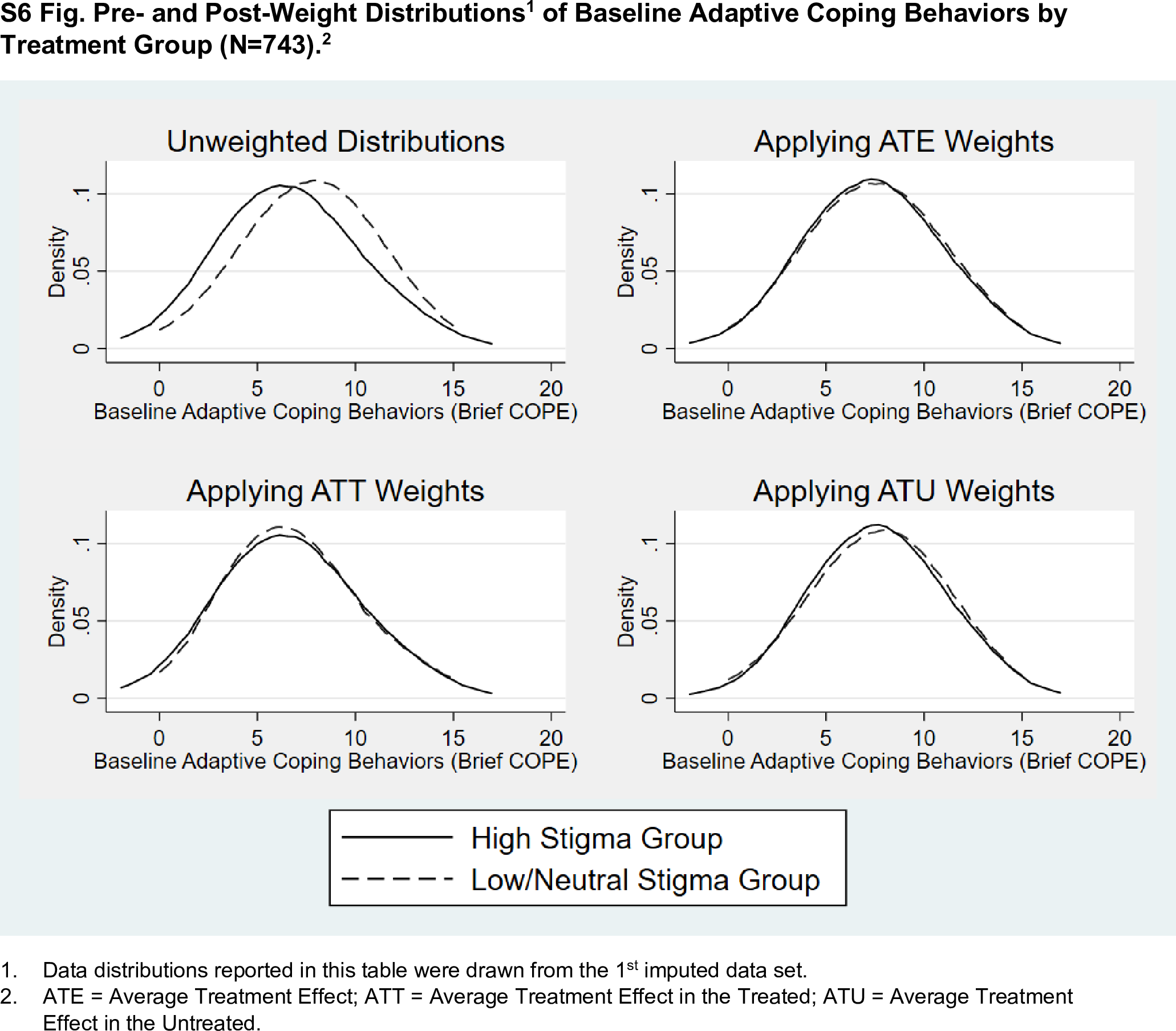

Supplement: S1 Fig — Data distributions reported in this table were drawn from the 1st imputed data set. (TIF) [file pone.0282016.s007.tif]

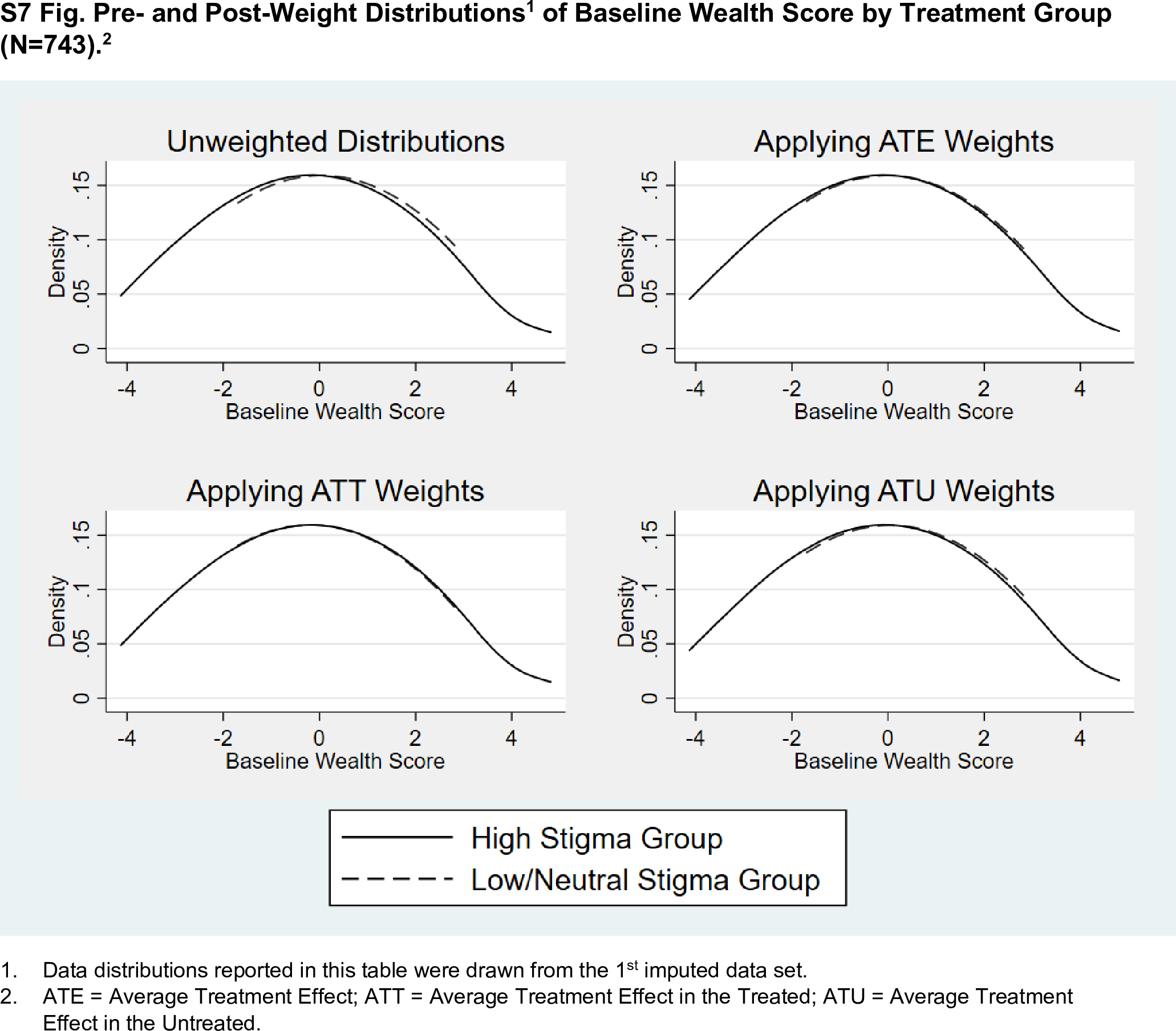

Supplement: S2 Fig — Data distributions reported in this table were drawn from the 1st imputed data set. (TIF) [file pone.0282016.s008.tif]

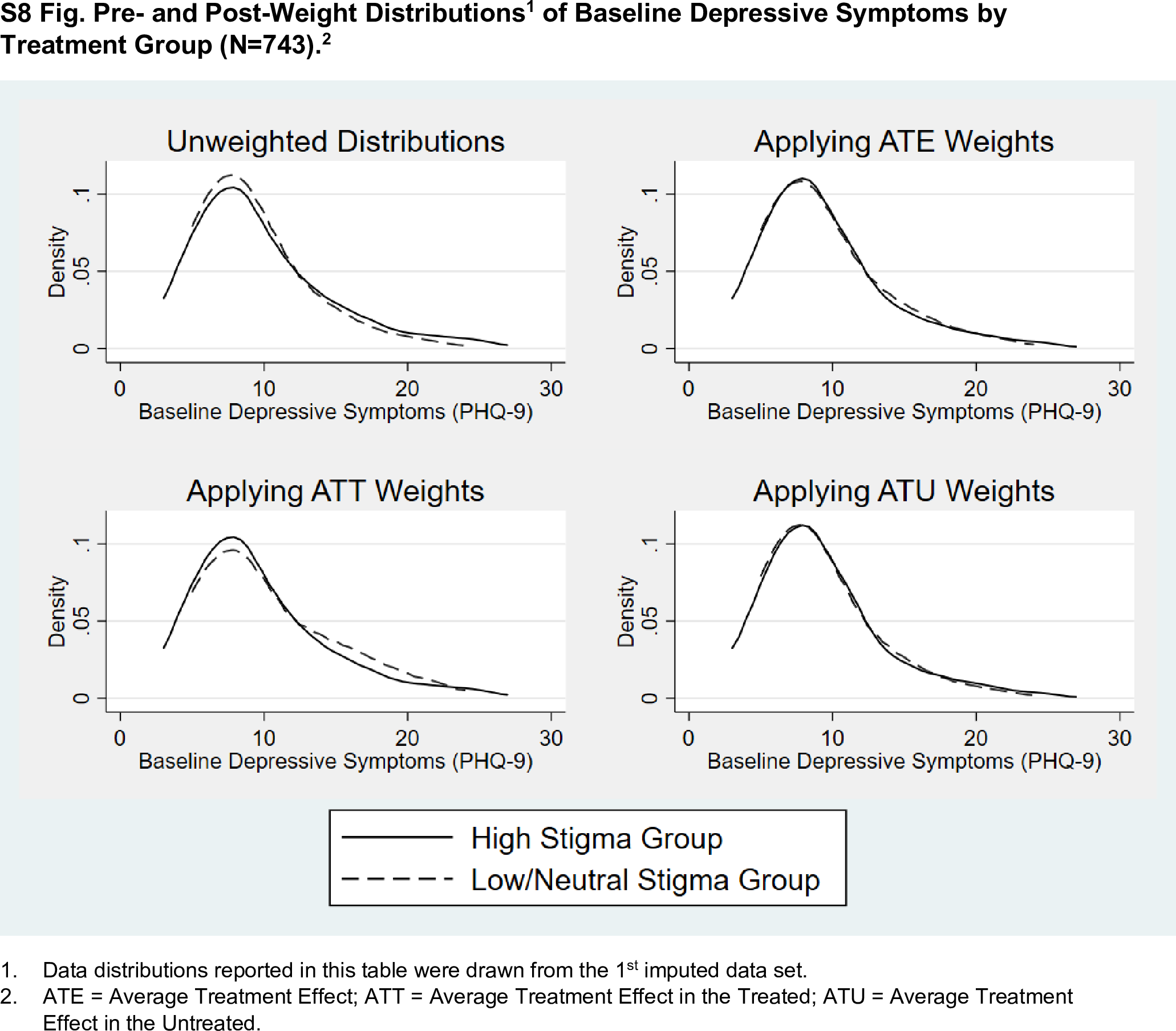

Supplement: S3 Fig — Data distributions reported in this table were drawn from the 1st imputed data set. (TIF) [file pone.0282016.s009.tif]

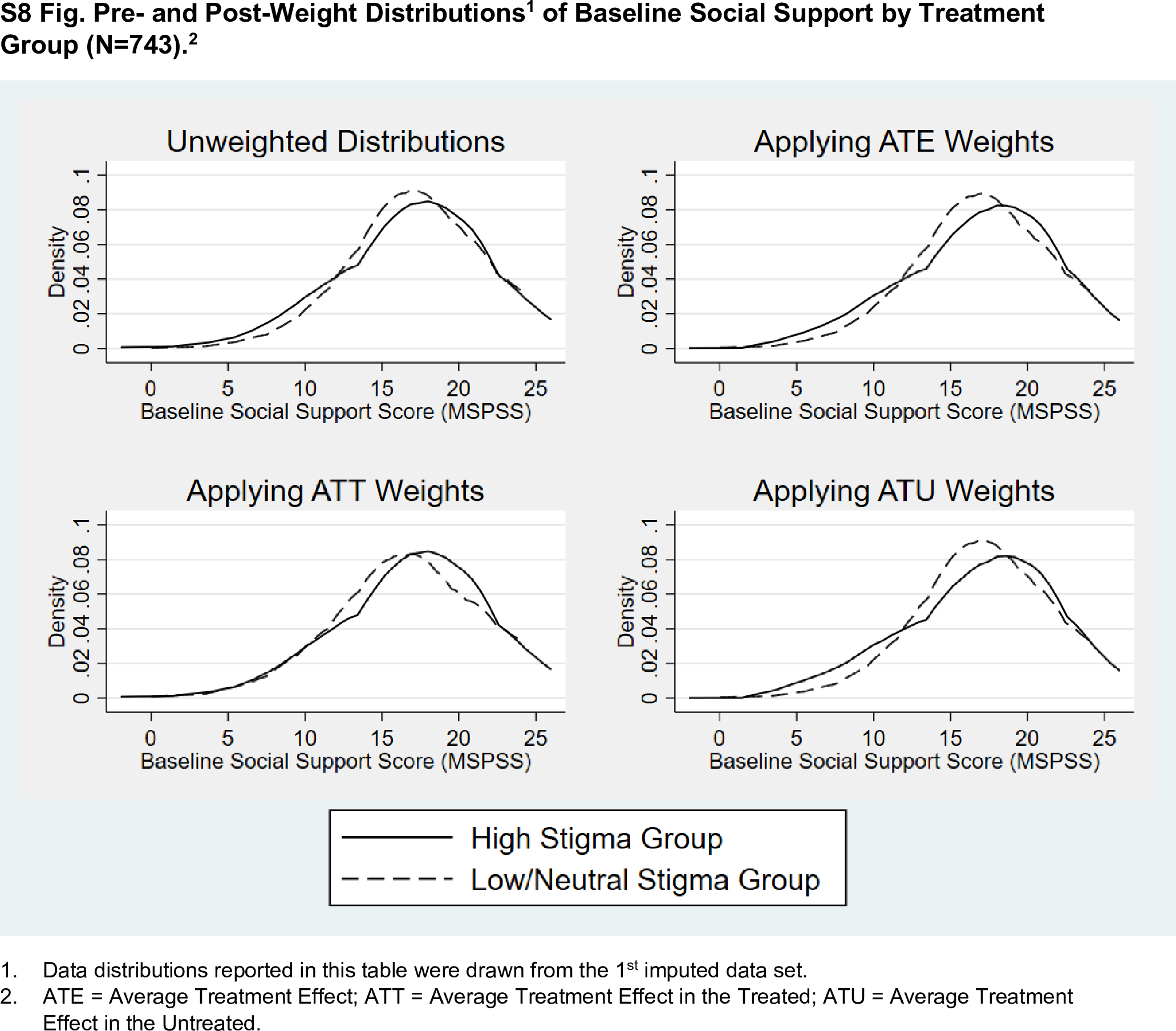

Supplement: S4 Fig — Data distributions reported in this table were drawn from the 1st imputed data set. (TIF) [file pone.0282016.s010.tif]
