# Supplementary material for: Estimating the effect of anticipated depression treatment-related stigma on depression remission among people with noncommunicable diseases and depressive symptoms in Malawi
Source: PLoS One. 2023 Mar 16;18(3):e0282016. doi: 10.1371/journal.pone.0282016 (PMC10019662; doi:10.1371/journal.pone.0282016)
Supplement: Attachment — Submitted filename: Response to Reviewers.docx [file pone.0282016.s011.docx]

**Response to Reviewers**

**We thank the reviewer for their time and thoughtful consideration of our manuscript. Please see our line-by-line responses (in bold) below with reference to where the improvements in the manuscript have been made.**

*Reviewer #1:* This study explored the role of treatment-related stigma in depression care by estimating the effect of patients’ baseline anticipated treatment-related stigma on their 3-month probability of depression remission when newly identified with depression.
This work makes important contributions to the literature regarding the public mental health issues.
However, the manuscript can be further strengthened by some reframing of the introduction, addition of further details regarding the methods, and clarification of study findings. I have provided additional details below.

**Thank you.**

Since the participants in this study are all patients with chronic diseases, it is suggested that the author should make it clear in the title that this research group is NCD patients with depressive symptoms, rather than "people with depression" as currently described.

**Thank you for this suggestion. We have updated the title to reflect this: “Estimating the effect of anticipated depression treatment-related stigma on depression remission among people with noncommunicable diseases and depressive symptoms in Malawi”**

*Abstract:*
It should be clarified that this study is based on cohort study design.

**Thank you – this has been added (line 8).**

In methods of abstract, the author stated that this study measured the data at four time points, but only analyzed the data after the third month in the text, which makes readers confused. I can understand that the data of this manuscript comes from a large research project and the observation at four time points is the design of the parent study. If the data in this manuscript does not use the follow-up data at the last two time points, it is recommended to modify this description in the abstract to avoid misunderstanding.

**Thank you for this note – it has been amended to avoid confusion (lines 10-11).**

*Introduction:*
There are some descriptions of the limitations of existing literature around mental illness stigma. However, the rationale for studying this topic in Malawi specifically could be bolstered.

**Thank you, this has been noted, and we have added further detail on the timeliness of this work in Malawi on lines 51-53.**

Specifically, additional support is needed for why you explored a series of confounding factors in method section (P9, line 131-159)? The main confounding factors considered in this study should be justified in the background.

**Thank you, we have provided more context in the background (lines 57-61) and methods (lines 137-151).**

*Methods:*
Interesting that the author considered multiple psychosocial indicators as confounding factors, but did not mention participants' chronic diseases, such as NCD condition and other physical health related characteristics. You could link this more clearly to the limitations section which discusses the overall healthiness of your study population.

**We have clarified our decision-making for confounder selection in the methods section (lines 137-151).**

Please omit the following sentences, “In general, this sample of participants had high levels of baseline social support and post-traumatic stress symptoms (P12, line 28-219).” And instead describe the actual findings presented in Table 1.

**In lieu of omitting the sentence, we have added the actual findings from the table into the sentence (lines 230-232). We have also added the scale ranges to Table 1 for greater intelligibility.**

Moreover, because the author did not describe the assessment method of social support and PTSD symptoms in method section, there is no basis for how to judge the "high level" here.

**Thank you. Please see the methods section, where we have cited the Multidimensional Scale of Perceived Social Support (MSPSS; lines 146-147) and the PTSD checklist (PCL-C; line 178). In Table 1, each scale’s range is now listed for ease of interpretation.**

*Result*:
The presentation of Table 2 is a bit confusing. It is suggested that the author present demographic characteristic variables together. Then it can be considered to put the observed variables after the baseline and three-month follow-up into a table and list them by two columns for comparison.

**We have created the table you suggested, combining the information from the previous 2 tables, and it is now labeled Table 1.**

I think Table S3 can present the main results better than Table 3.

**We have moved Table S3 to the main text, and it is now labeled Table 2. We have re-numbered all supplementary material accordingly.**

*Discussion*:
As the author stated (in the methodology section), the stigma experienced by the participants was different from the anticipated stigma. However, I didn't see the author mention in the discussion what kind of targeted intervention should be adopted against the “anticipated treatment-related stigma” in this study. And, whether it is necessary to design and intervene the content of this type of stigma from the perspective of patients themselves, rather than reducing the discriminatory attitude towards community people in general? Please clarify.

**We fully agree that this is an important matter to address. Please see paragraph 2 of the discussion section.**
